# Supplementary figures and images for: The chemorepellent, SLIT2, bolsters innate immunity against Staphylococcus aureus
Source: eLife. 2023 Sep 29;12:e87392. doi: 10.7554/eLife.87392 (PMC10541174; doi:10.7554/eLife.87392)

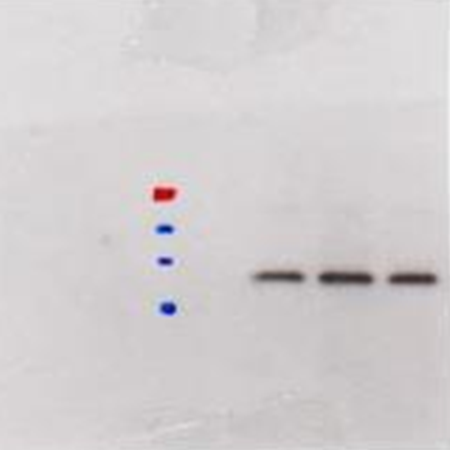

Supplement: Figure 2—source data 2. [file elife-87392-fig2-data2.zip › Figure2_SourceData2/Figure 2 - Unlabled blots/Figure 2C p38 MAPK_UL.tif]

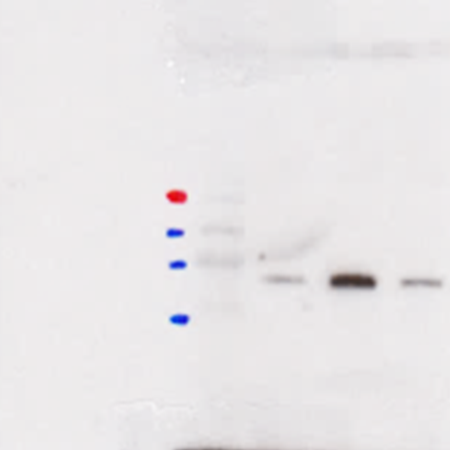

Supplement: Figure 2—source data 2. [file elife-87392-fig2-data2.zip › Figure2_SourceData2/Figure 2 - Unlabled blots/Figure 2C phospho-p38 MAPK_UL.tif]

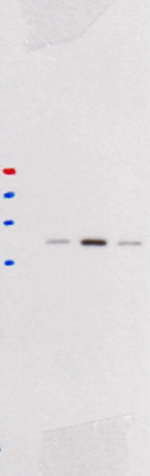

Supplement: Figure 2—source data 2. [file elife-87392-fig2-data2.zip › Figure2_SourceData2/Figure 2 - Unlabled blots/Figure 2G phospho-p38 MAPK_UL.tif]

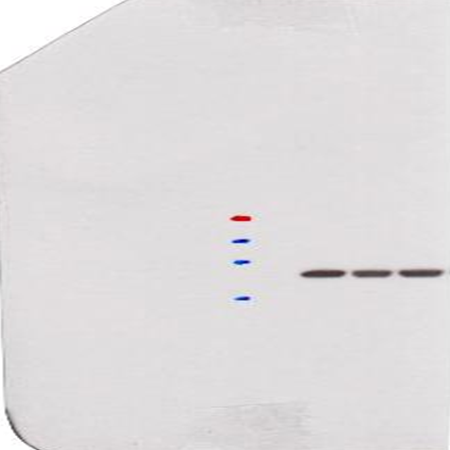

Supplement: Figure 2—source data 2. [file elife-87392-fig2-data2.zip › Figure2_SourceData2/Figure 2 - Unlabled blots/Figure 2A NCF1_UL.tif]

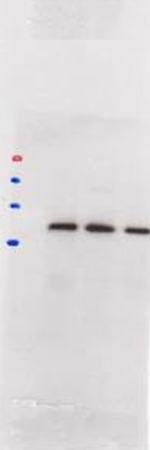

Supplement: Figure 2—source data 2. [file elife-87392-fig2-data2.zip › Figure2_SourceData2/Figure 2 - Unlabled blots/Figure 2G p38 MAPK_UL.tif]

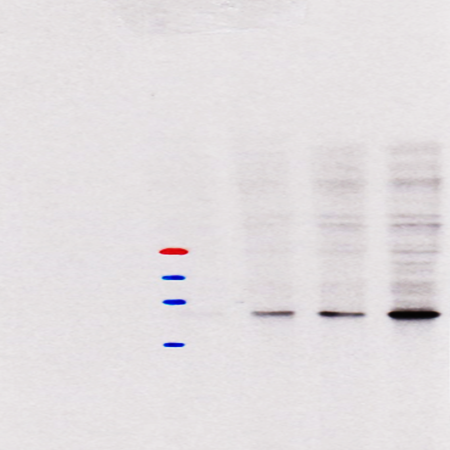

Supplement: Figure 2—source data 2. [file elife-87392-fig2-data2.zip › Figure2_SourceData2/Figure 2 - Unlabled blots/Figure 2A phospho-NCF1_UL.tif]

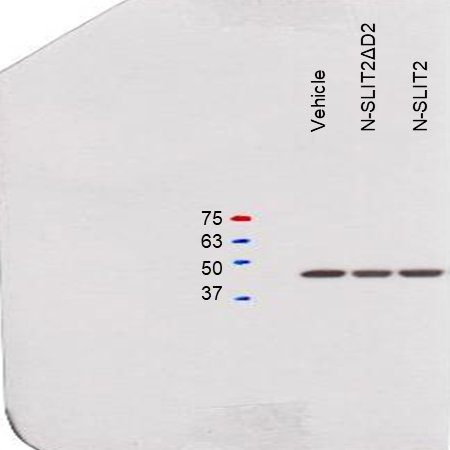

Supplement: Figure 2—source data 2. [file elife-87392-fig2-data2.zip › Figure2_SourceData2/Figure 2 - Labled blots/Figure 2A NCF1.tif]

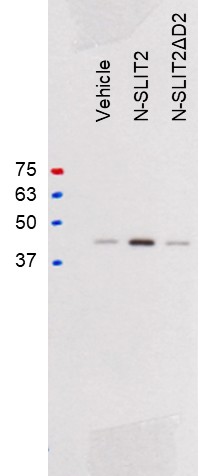

Supplement: Figure 2—source data 2. [file elife-87392-fig2-data2.zip › Figure2_SourceData2/Figure 2 - Labled blots/Figure 2G phospho-p38 MAPK.tif]

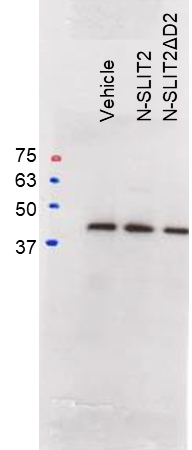

Supplement: Figure 2—source data 2. [file elife-87392-fig2-data2.zip › Figure2_SourceData2/Figure 2 - Labled blots/Figure 2G p38 MAPK.tif]

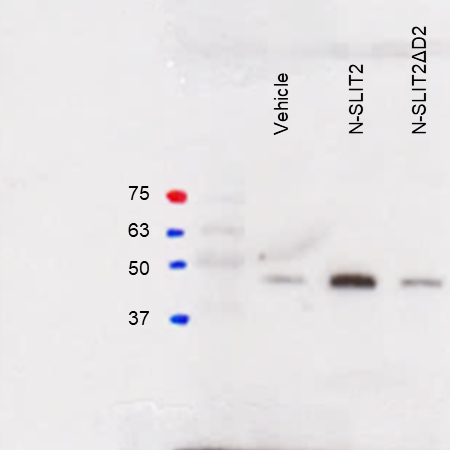

Supplement: Figure 2—source data 2. [file elife-87392-fig2-data2.zip › Figure2_SourceData2/Figure 2 - Labled blots/Figure 2C phospho-p38 MAPK.tif]

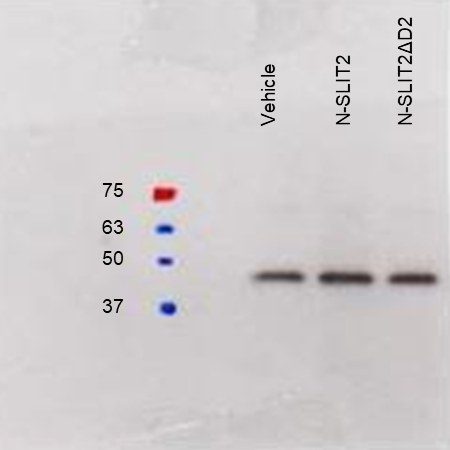

Supplement: Figure 2—source data 2. [file elife-87392-fig2-data2.zip › Figure2_SourceData2/Figure 2 - Labled blots/Figure 2C p38 MAPK.tif]

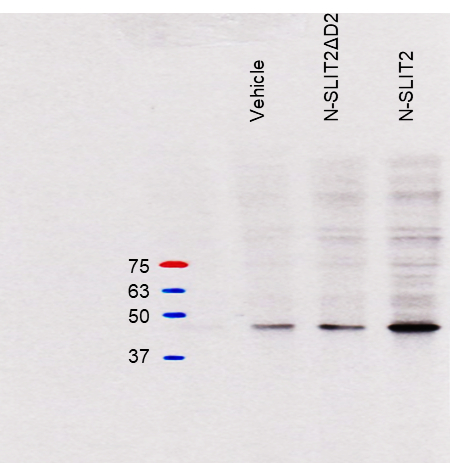

Supplement: Figure 2—source data 2. [file elife-87392-fig2-data2.zip › Figure2_SourceData2/Figure 2 - Labled blots/Figure 2A phospho-NCF1.tif]

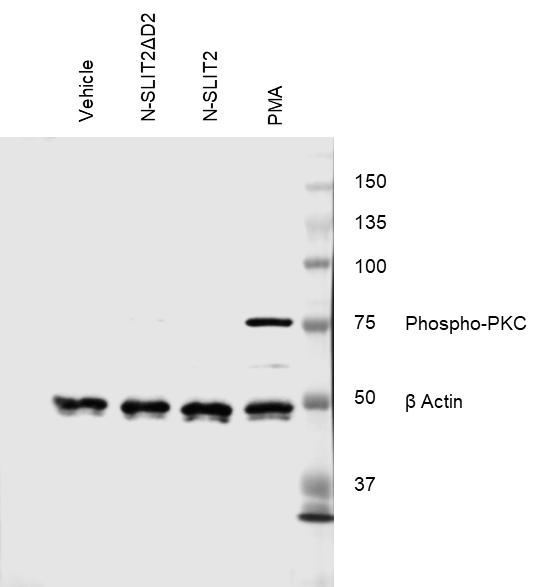

Supplement: Figure 2—figure supplement 1—source data 2. [file elife-87392-fig2-figsupp1-data2.zip › Figure2FigureSupplement1_SourceData2/Figure 2 - Figure Supplement 1 - Labeled blots/Figure 2 figure supplement 1C.tif]

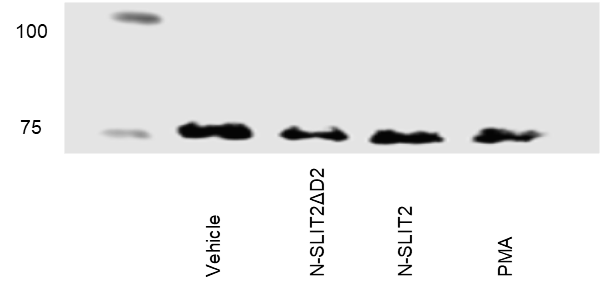

Supplement: Figure 2—figure supplement 1—source data 2. [file elife-87392-fig2-figsupp1-data2.zip › Figure2FigureSupplement1_SourceData2/Figure 2 - Figure Supplement 1 - Labeled blots/Figure 2 figure supplement 1E PKC (delta).tif]

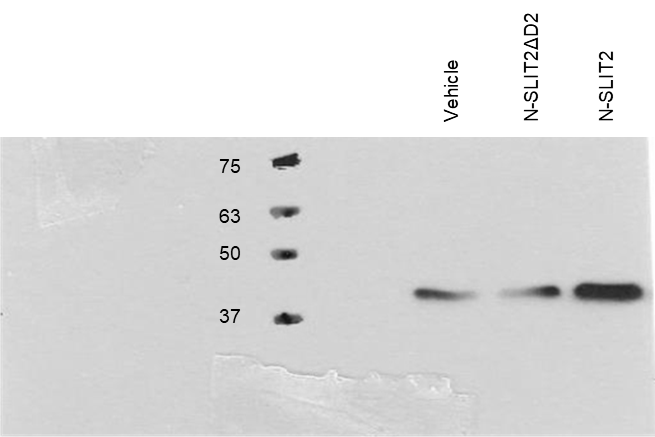

Supplement: Figure 2—figure supplement 1—source data 2. [file elife-87392-fig2-figsupp1-data2.zip › Figure2FigureSupplement1_SourceData2/Figure 2 - Figure Supplement 1 - Labeled blots/Figure 2 figure supplement 1G phospho-p38 MAPK.tif]

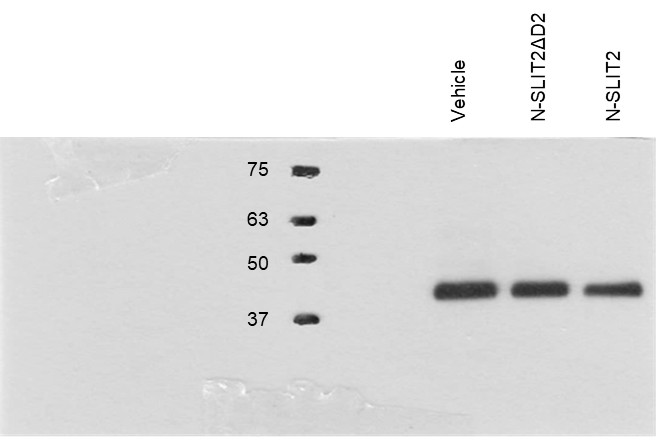

Supplement: Figure 2—figure supplement 1—source data 2. [file elife-87392-fig2-figsupp1-data2.zip › Figure2FigureSupplement1_SourceData2/Figure 2 - Figure Supplement 1 - Labeled blots/Figure 2 figure supplement 1G p38 MAPK.tif]

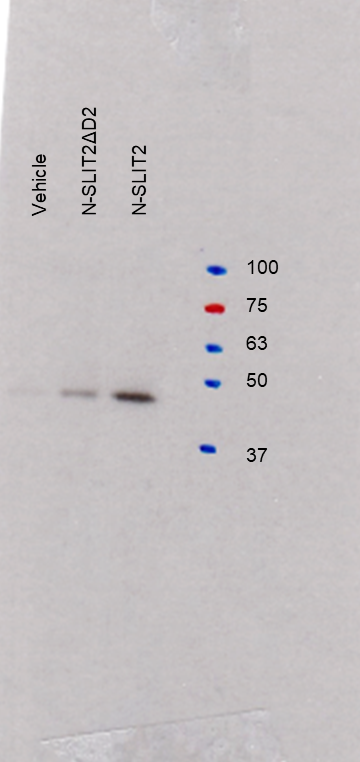

Supplement: Figure 2—figure supplement 1—source data 2. [file elife-87392-fig2-figsupp1-data2.zip › Figure2FigureSupplement1_SourceData2/Figure 2 - Figure Supplement 1 - Labeled blots/Figure 2 figure supplement 1A phospho-NCF1.tif]

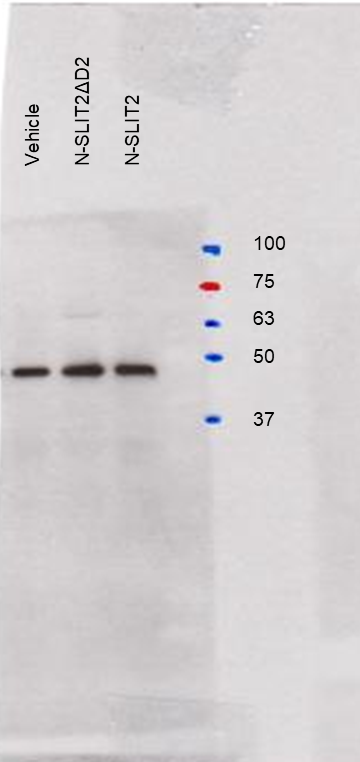

Supplement: Figure 2—figure supplement 1—source data 2. [file elife-87392-fig2-figsupp1-data2.zip › Figure2FigureSupplement1_SourceData2/Figure 2 - Figure Supplement 1 - Labeled blots/Figure 2 figure supplement 1A NCF1.tif]

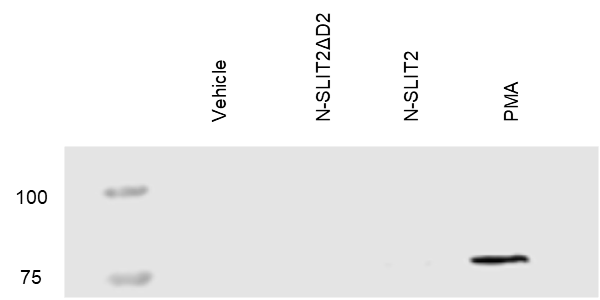

Supplement: Figure 2—figure supplement 1—source data 2. [file elife-87392-fig2-figsupp1-data2.zip › Figure2FigureSupplement1_SourceData2/Figure 2 - Figure Supplement 1 - Labeled blots/Figure 2 figure supplement 1E phospho-PKC (delta).tif]

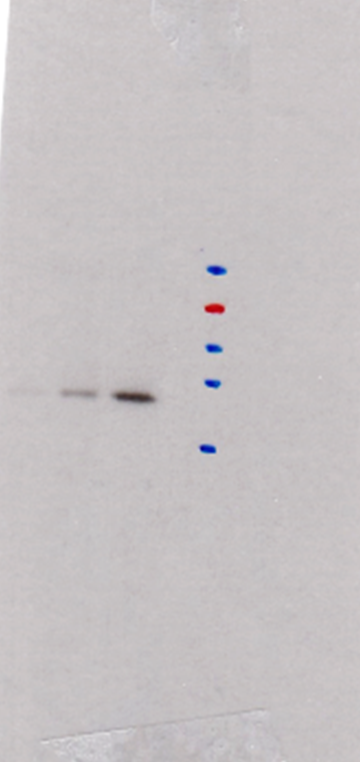

Supplement: Figure 2—figure supplement 1—source data 2. [file elife-87392-fig2-figsupp1-data2.zip › Figure2FigureSupplement1_SourceData2/Figure 2 - Figure Supplement 1 - Unlabeled blots/Figure 2 figure supplement 1A phospho-NCF1_UL.tif]

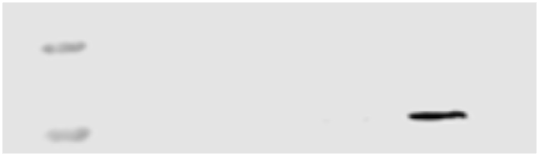

Supplement: Figure 2—figure supplement 1—source data 2. [file elife-87392-fig2-figsupp1-data2.zip › Figure2FigureSupplement1_SourceData2/Figure 2 - Figure Supplement 1 - Unlabeled blots/Figure 2 figure supplement 1E phospho-PKC (delta)_UL.tif]

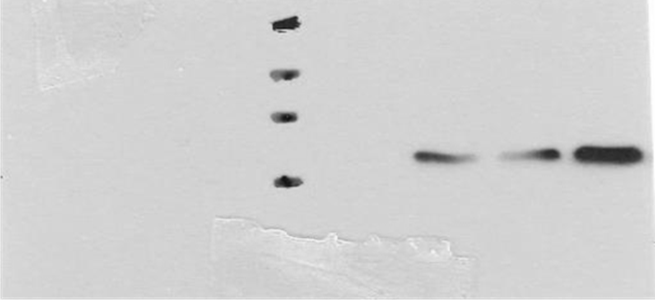

Supplement: Figure 2—figure supplement 1—source data 2. [file elife-87392-fig2-figsupp1-data2.zip › Figure2FigureSupplement1_SourceData2/Figure 2 - Figure Supplement 1 - Unlabeled blots/Figure 2 figure supplement 1G phospho-p38 MAPK_UL.tif]

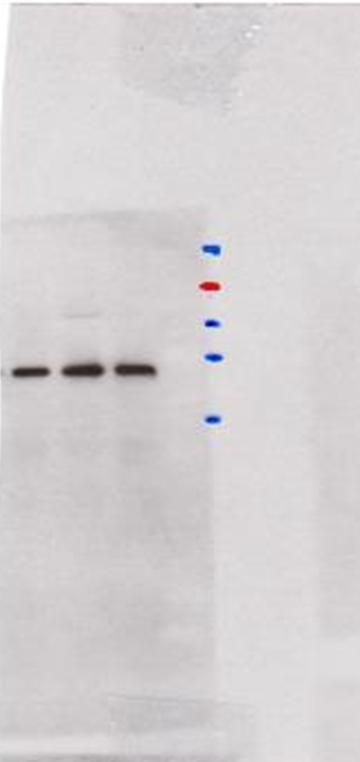

Supplement: Figure 2—figure supplement 1—source data 2. [file elife-87392-fig2-figsupp1-data2.zip › Figure2FigureSupplement1_SourceData2/Figure 2 - Figure Supplement 1 - Unlabeled blots/Figure 2 figure supplement 1A NCF1_UL.tif]

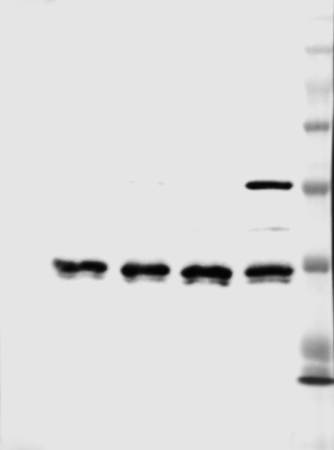

Supplement: Figure 2—figure supplement 1—source data 2. [file elife-87392-fig2-figsupp1-data2.zip › Figure2FigureSupplement1_SourceData2/Figure 2 - Figure Supplement 1 - Unlabeled blots/Figure 2 figure supplement 1C_UL.tif]

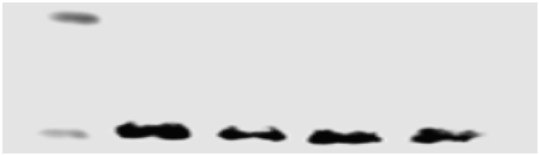

Supplement: Figure 2—figure supplement 1—source data 2. [file elife-87392-fig2-figsupp1-data2.zip › Figure2FigureSupplement1_SourceData2/Figure 2 - Figure Supplement 1 - Unlabeled blots/Figure 2 figure supplement 1E PKC (delta)_UL.tif]

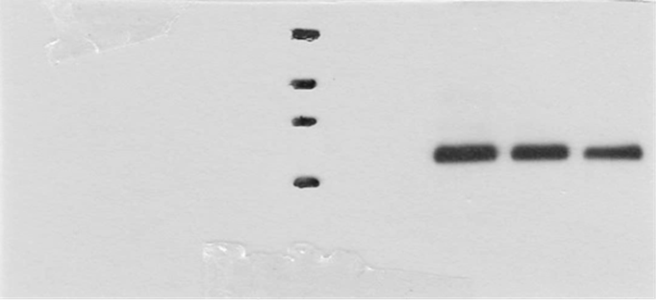

Supplement: Figure 2—figure supplement 1—source data 2. [file elife-87392-fig2-figsupp1-data2.zip › Figure2FigureSupplement1_SourceData2/Figure 2 - Figure Supplement 1 - Unlabeled blots/Figure 2 figure supplement 1G p38 MAPK_UL.tif]
